# Supplementary material for: Vitamin D Modified DSS-Induced Colitis in Mice via STING Signaling Pathway
Source: Biology (Basel). 2025 Jun 18;14(6):715. doi: 10.3390/biology14060715 (PMC12190092; doi:10.3390/biology14060715)
Supplement: Supplementary file 1 [file biology-14-00715-s001.zip › Supplementary Materials S1: 16s-report of gut microbiota/Groups/diff_analysis/TaxaMarkers/Family.Groups.sig.boxplot.pdf]

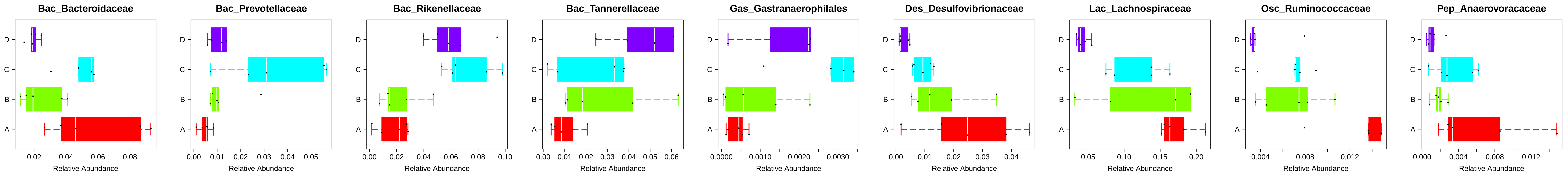
Bac_Bacteroidaceae
Bac_Prevotellaceae
Bac_Rikenellaceae
Bac_Tannerellaceae
Gas_Gastranaerophilales
Des_Desulfovibrionaceae
Lac_Lachnospiraceae
Osc_Ruminococcaceae
Pep_Anaerovoracaceae
D
C
B
A
Relative Abundance
